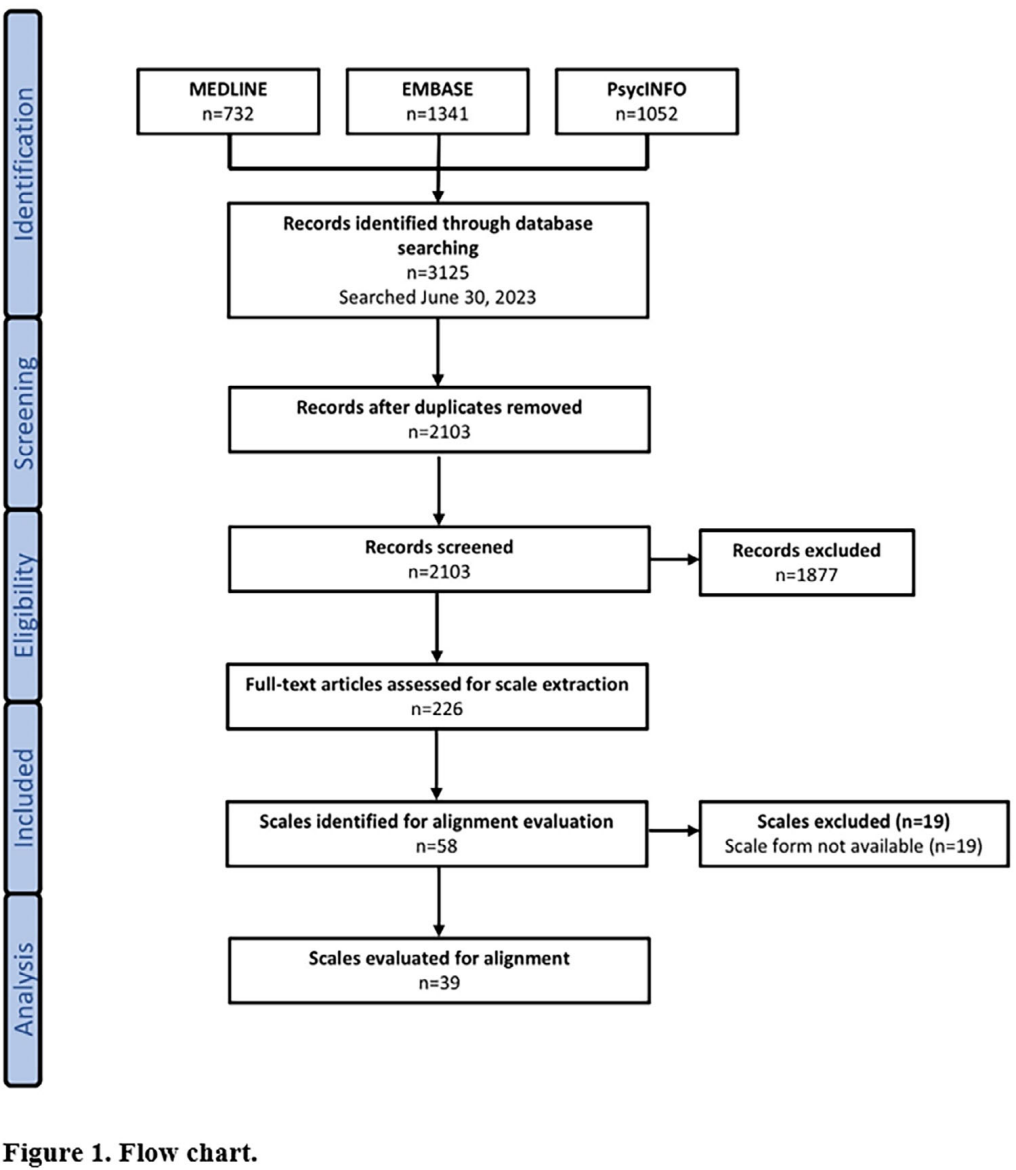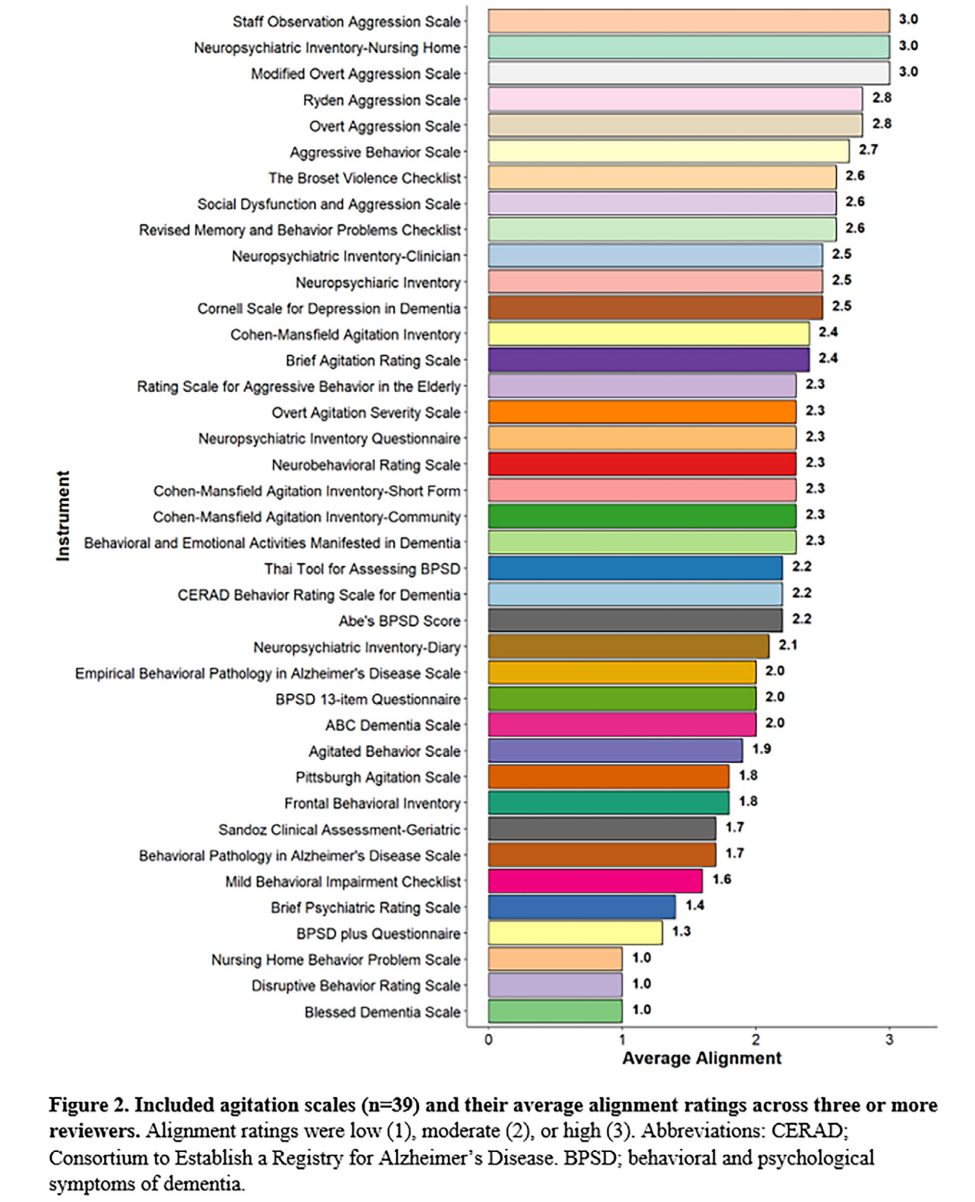# The measurement of agitation in neurocognitive disorders: A systematic review

**DOI:** 10.1002/alz70857_105072

**Published:** 2025-12-25

**Authors:** Dylan X. Guan, Byron Creese, Cassandra M Germain, Corinne E. Fischer, Dorota Kossowska‐Kuhn, Fabricio Ferreira de Oliveira, Hui Jue (Janet) Wang, Keerthana Umapathy, Krista L Lanctôt, Lindsey I Sinclair, Luis Agüera‐Ortiz, Mariana Pinheiro Nepomuceno, Marie‐Andrée Bruneau, Moyra E Mortby, Ram J Bishnoi, Raphael Machado Castilhos, Reeteka Sud, Samantha M Loi, Soliu A. Atunwa, Tiffany J Pan, Toni T Saari, Uzochukwu Imo, Valentine A Ucheagwu, Zahinoor Ismail, Alzheimer's Association International Society to Advance Alzheimer's Research and Treatment (ISTAART) Agitation work group

**Affiliations:** ^1^ Hotchkiss Brain Institute, University of Calgary, Calgary, AB, Canada; ^2^ Brunel University of London, London, United Kingdom; ^3^ North Carolina A&T State University, Greensboro, NC, USA; ^4^ Keenan Research Centre for Biomedical Science, Li Ka Shing Knowledge Institute, St. Michael's Hospital, Toronto, ON, Canada; ^5^ Florida State University, Tallahassee, FL, USA; ^6^ Federal University of São Paulo ‐ UNIFESP, São Paulo, São Paulo, Brazil; ^7^ Sunnybrook Research Institute, Toronto, ON, Canada; ^8^ University of Toronto, Toronto, ON, Canada; ^9^ Queen's University Belfast, Belfast, Northern Ireland, United Kingdom; ^10^ National Institute of Mental Health and Neurosciences [NIMHANS], Bengaluru, Karnataka, India; ^11^ University of Bristol, Bristol, United Kingdom; ^12^ University Hospital 12 de Octubre, Madrid, Spain; ^13^ University of Valencia, Valencia, Spain; ^14^ University of Montreal, Montreal, QC, Canada; ^15^ Institut Universitaire de Gériatrie de Montréal Research Center, Montréal, QC, Canada; ^16^ Neuroscience Research Australia, Sydney, NSW, Australia; ^17^ UNSW Ageing Futures Institute, Sydney, NSW, Australia; ^18^ University of New South Wales, Sydney, NSW, Australia; ^19^ USF Health Byrd Alzheimer's Institute, Tampa, FL, USA; ^20^ Hospital de Clínicas de Porto Alegre, Porto Alegre, Rio Grande do Sul, Brazil; ^21^ ADBS Lab, National Institute of Mental Health and Neurosciences (NIMHANS), Bengaluru, Karnataka, India; ^22^ University of Melbourne, Parkville, VIC, Australia; ^23^ Neuropsychiatry, The Royal Melbourne Hospital, Parkville, VIC, Australia; ^24^ University of Ilorin, Ilorin, Nigeria; ^25^ Obafemi Awolowo University, Ile‐Ife, Nigeria; ^26^ University of Helsinki, Helsinki, Finland; ^27^ R‐Jolad Hospital, Lagos, Lagos, Nigeria; ^28^ Nnamdi Azikiwe University Awka, Onitsha / Anambra State, Nigeria

## Abstract

**Background:**

Agitation is a common and distressing behavior in persons with neurocognitive disorders. However, efforts to understand and develop interventions for agitation, historically considered only as a symptom, have been complicated by heterogeneity in the definition, identification, and measurement of agitation. The International Psychogeriatric Association (IPA) developed and then validated a consensus clinical and research syndromic definition of agitation in cognitive disorders. The Neuropsychiatric Syndromes Professional Interest Area Agitation Work Group conducted a systematic review to identify validated measures of agitation used in older persons with neurocognitive disorders and to evaluate their alignment with IPA criteria.

**Method:**

This review was pre‐registered on PROSPERO (CRD42023429494). We searched MEDLINE, EMBASE, and PsycINFO from inception to June 30, 2023, using a search strategy that included term clusters for 1) neurocognitive disorders; 2) agitation; and 3) psychometric outcomes. Title/abstract screening was performed to include validation studies of original agitation scales in populations with neurocognitive disorders (e.g., mild cognitive impairment, dementia). The full texts of these studies were then reviewed to extract agitation scales. Scale instructions, items, and response fields for each scale were evaluated for alignment with IPA agitation criteria by at least three independent reviewers.

**Result:**

A total of 2103 unique search records were retrieved, of which 1877 were excluded at title/abstract screening. From the 226 full‐text articles, 39 unique agitation scales were identified. The three scales containing agitation items that showed the greatest alignment with IPA criteria were the Staff Observation Aggression Scale, Neuropsychiatric Inventory Nursing Home, and Modified Overt Aggression Scale. Although all 39 scales included at least one item measuring verbal aggression, eight scales did not address excessive motor activity, and five scales did not include items for physical aggression.

**Conclusion:**

Numerous agitation scales have been validated in older populations with neurocognitive disorders. Yet, few fully align with the recently published IPA agitation criteria. Most commonly, scales did not adequately capture symptom persistence of at least two weeks or evidence of emotional distress. Additionally, scales varied widely in capturing individual IPA agitation domains of verbal aggression, excessive motor activity, and physical aggression.